# A new design of a lentiviral shRNA vector with inducible co-expression of ARGONAUTE 2 for enhancing gene silencing efficiency

**DOI:** 10.1186/s13578-015-0058-2

**Published:** 2015-12-08

**Authors:** Jiening He, Lian Huang, Huiling Qiu, Jiexuan Li, Lan Luo, Yanjiao Li, Shengli Tian, Kang Kang, Jun Luo, Lin Liu, Deming Gou

**Affiliations:** Shenzhen Key Laboratory of Microbial Genetic Engineering, Shenzhen Key Laboratory of Marine Bioresource and Eco-environmental Science, College of Life Sciences, Shenzhen University, Nanhai Ave 3688, Shenzhen, 518060 Guangdong China; Department of Physiology, Shenzhen University Health Science Center, Shenzhen University, Shenzhen, 518060 Guangdong China; College of Animal Science and Technology, Northwest A&F University, Yangling, 712100 Shaanxi China; Department of Physiological Sciences, Oklahoma State University, Stillwater, OK 74078 USA

**Keywords:** RNAi, shRNA, AGO2, Lentivirus

## Abstract

**Background:**

RNA interference (RNAi) is a robust tool for inhibiting specific gene expression, but it is limited by the uncertain efficiency of siRNA or shRNA constructs. It has been shown that the overexpression of ARGONAUTE 2 (AGO2) protein increases silencing efficiency. However, the key elements required for AGO2-mediated enhancement of gene silencing in lentiviral vector has not been well studied.

**Results:**

To explore the application of AGO2-based shRNA system in mammalian cells, we designed shRNA vectors targeting the EGFP reporter gene and evaluated the effects of various factors on silencing efficiency including stem length, loop sequence, antisense location as well as the ratio between AGO2 and shRNA. We found that 19 ~ 21-bp stem and 6- or 9-nt loop structure in the sense-loop-antisense (S-L-AS) orientation was an optimal design in the AGO2-shRNA system. Then, we constructed a single lentiviral vector co-expressing shRNA and AGO2 and demonstrated that the simultaneous expression of shRNA and AGO2 can achieve robust silencing of exogenous DsRed2 and endogenous ID1 and P65 genes. However, the titers of packaged lentivirus from constitutive expression of AGO2 vector were extremely low, severely limiting its broad application. For the first time, we demonstrated that the problem can be significantly improved by using the inducible expression of AGO2 lentiviral system.

**Conclusions:**

We reported a novel lentiviral vector with an optimal design of shRNA and inducible AGO2 overexpression which provides a new tool for RNAi research.

## Background

Over one and a half decades after RNA inference (RNAi) discovery in nematodes [1], RNAi-based technology (e.g. siRNA and shRNA) is now widely used for gene knockdown in basic scientific research and provides promise to new therapeutic strategies for a variety of diseases [2, 3]. With the development of siRNA or shRNA libraries, and high-throughput screening techniques, genome-wide RNAi screening is employed for broad and unbiased identification of genetic modifiers of various cell- or organism-based phenotypes [4, 5].

In principle, RNAi can be used to repress the expression of any genes. However, it is still much unknown about the siRNA biogenesis and action of mechanism. The application of RNAi technique is limited by the inconsistent efficiency of targeting sequences [6]. The existing computational approaches for siRNA selection allow the identification of potential sequences, but they do not ensure that each selected siRNA has sufficient silencing efficiency. It has been observed that many designed siRNAs or shRNAs are not effective. On average, about 25 % of the selected siRNA/shRNA sequences are functional with a knockdown efficiency more than 75 %. As a result, it is recommended to screen highly potent siRNA/shRNA sequences from at least four potential sites of a given mRNA [7]. Therefore, an approach to enhance RNAi efficiency is desired for its broad applications in gene-function analysis, drug-target discovery and validation, and developing therapeutic siRNA/shRNA for various diseases.

Several attempts have been made to improve RNAi efficacy. For example, modifications can be made to improve the stability of chemically synthesized siRNA, resulting in enhanced siRNA inhibition efficacy [8]. For vector-based shRNA, improvements can be achieved through manipulating expression strategy, using different types of promoters, modifying shRNA structures, and simultaneously expressing multiple shRNAs in a single vector [9–13]. Another strategy was developed to enhance siRNA biogenesis using the small molecule, enoxacin. Importantly, this molecule has only minor effects on cellular gene or miRNA expression [14].

Over the last few years, a better understanding of RNAi mechanism facilitates the identification of key proteins involved in the RNAi pathway, including the members of the ARGONAUTE family, DICER, EXPORTIN-5 (XPO5) and trp RNA-binding attenuation protein (TRAP) [15, 16]. Several groups have demonstrated that co-expression of some key components with shRNAs increases the RNAi efficacy [6, 17–19]. For example, overexpression of XPO5, a factor required for nuclear export of both shRNAs and pre-miRNAs, enhances RNAi mediated by shRNAs [17]. Similarly, overexpressing AGO2 also increases efficiency of siRNA or shRNA-mediated gene silencing in vitro and in vivo [6, 19–22]. In this study, we evaluated key elements required for AGO2-mediated enhancement of gene silencing and developed a novel lentiviral shRNA vector with inducible co-expression of AGO2.

## Results

### Co-expression of AGO2 protein strongly enhances shRNA silencing activities

**Fig. 1:**
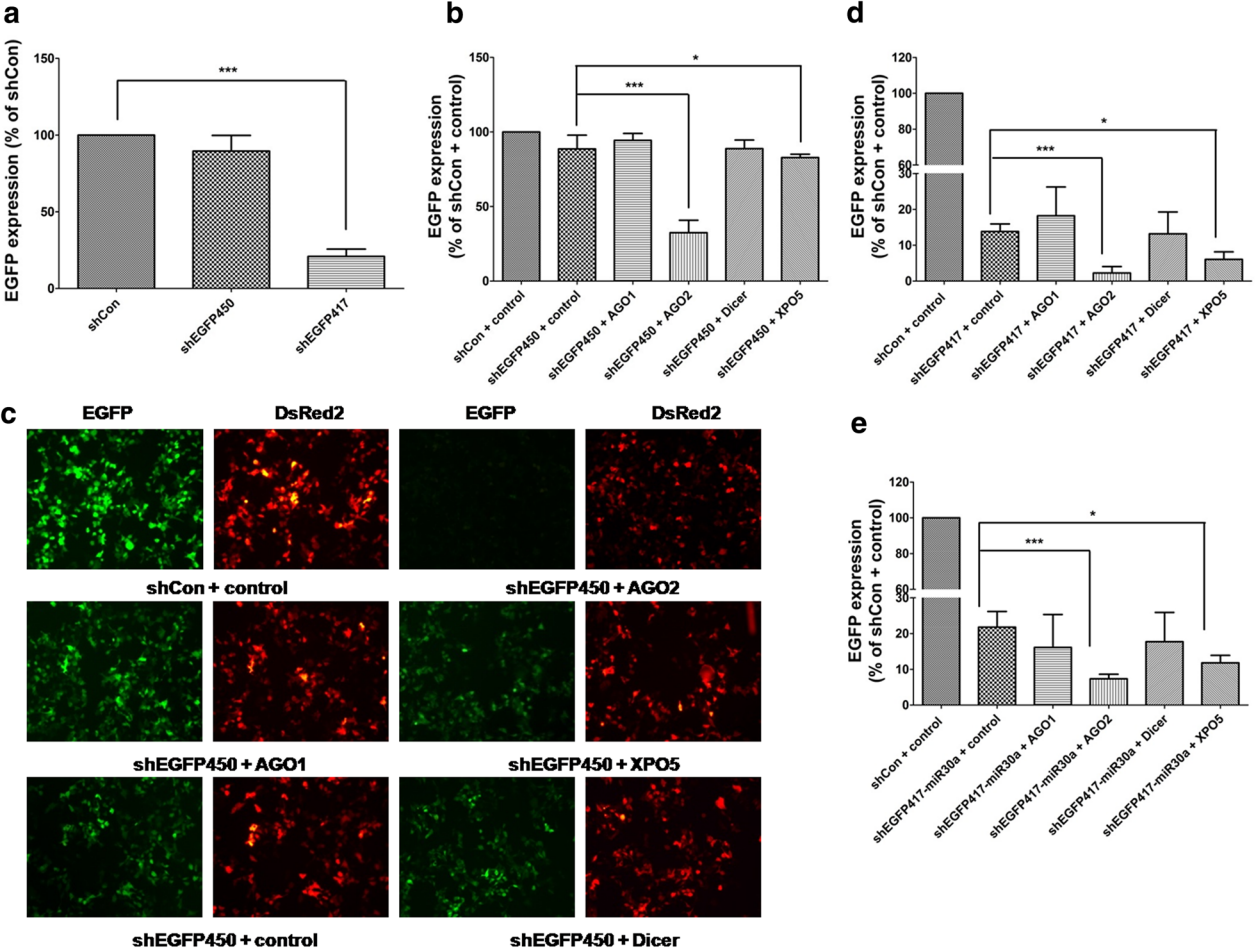
Effects of AGO1, AGO2, DICER or XPO5 overexpression on gene silencing. **a** Comparison of the silencing activities of two shRNAs against EGFP. HEK293 cells were co-transfected with pENTR/CMV-EGFP, pDsRed2-c1 and U6 promoter-driven shEGFP450, shEGFP417 or an unrelated shCon. Two days after transfection, the cells were then collected for flow cytometry analysis to evaluate EGFP knockdown efficiency by determining *green* to *red* (G/R) fluorescence intensity ratio; **b**, **c** effects of AGO1, AGO2, Dicer or XPO5 overexpression on the silencing of EGFP with shEGFP417 or shEGFP450. HEK293 cells were co-transfected with shRNA plasmid (shEGFP450, shEGFP417 or shCon), pENTR/CMV-EGFP, pDsRed2-c1 and plasmid expressing AGO1, AGO2, Dicer, XPO5 or empty control vector. Two days after transfection, G/R fluorescence intensity ratio was analyzed by flow cytometry and normalized to the shCon/control group; **d** representative images from the cells 2 days after transfection of pENTR/CMV-EGFP, pDsRed2-c1 and shGFP450 plasmids in the presence of AGO1, AGO2, Dicer, XPO5 or empty control vector; **e** G/R fluorescence intensity ratio measured from the cells transfected with shRNAmiR30a-based shEGFP417 (shEGFP417-miR30a) and the plasmid expressing AGO1, AGO2, Dicer, XPO5 or empty vector control. The results shown are mean ± standard deviation (SD) from three independent experiments. *P < 0. 05, ***P < 0.001

We first examined the efficiency of two shRNA sequences in silencing EGPF. U6 promoter-driven shEGFP417, shEGFP450 or an unrelated control (shCon) vector was co-transfected with pENTR/CMV-EGFP and pDsRed2-c1 plasmids into HEK293 cells. Two days after transfection, shEGFP417 exhibited an ~84 % knockdown efficiency compared to shCon, while shEGFP450 showed a weak silent activity (Fig. 1a).

To test whether the increase in protein levels of some crucial components in RNAi machinery would facilitate the silencing effect, we co-transfectd AGO1, AGO2, Dicer or XPO5 with shRNA vector into HEK293 cells and examined silencing efficiency. The results showed that AGO2 overexpression significantly enhanced the silencing activities of shEGFP417 from 84 % ± 2.1 to 96 % ± 1.8 and shEGFP450 from 12 % ± 9.2 to 70 % ± 4.4, respectively (Fig. 1b, c, d). However, overexpression of XPO5 showed merely a limited improvement of silencing activities compared to that of AGO2 (Fig. 1b, c). Overexpression of AGO1 and DICER did not show any enhancement in gene silencing.

Evidence from a large-scale study has indicated that miR-30a-based shRNAmiR30 vector produced a more effective silencing activity [23]. Therefore, we further examined the effects of these RNAi components on shRNAmiR30-based gene silencing by co-transfecting pSM2c-shEGFP417miR30 vector, EGFP/DsRed2-expressing plasmids and a vector overexpressing each RNAi component. Similar to the results using the U6-shRNA system, overexpression of AGO2 also showed the most significant improvement of silencing efficacy with the miR-30a-based shRNA vector (Fig. 1e). These data also suggested that AGO2 was a dominant factor facilitating miR-30a-based shRNA activities and likely to be a limiting factor for the efficient knockdown.

**Fig. 2:**
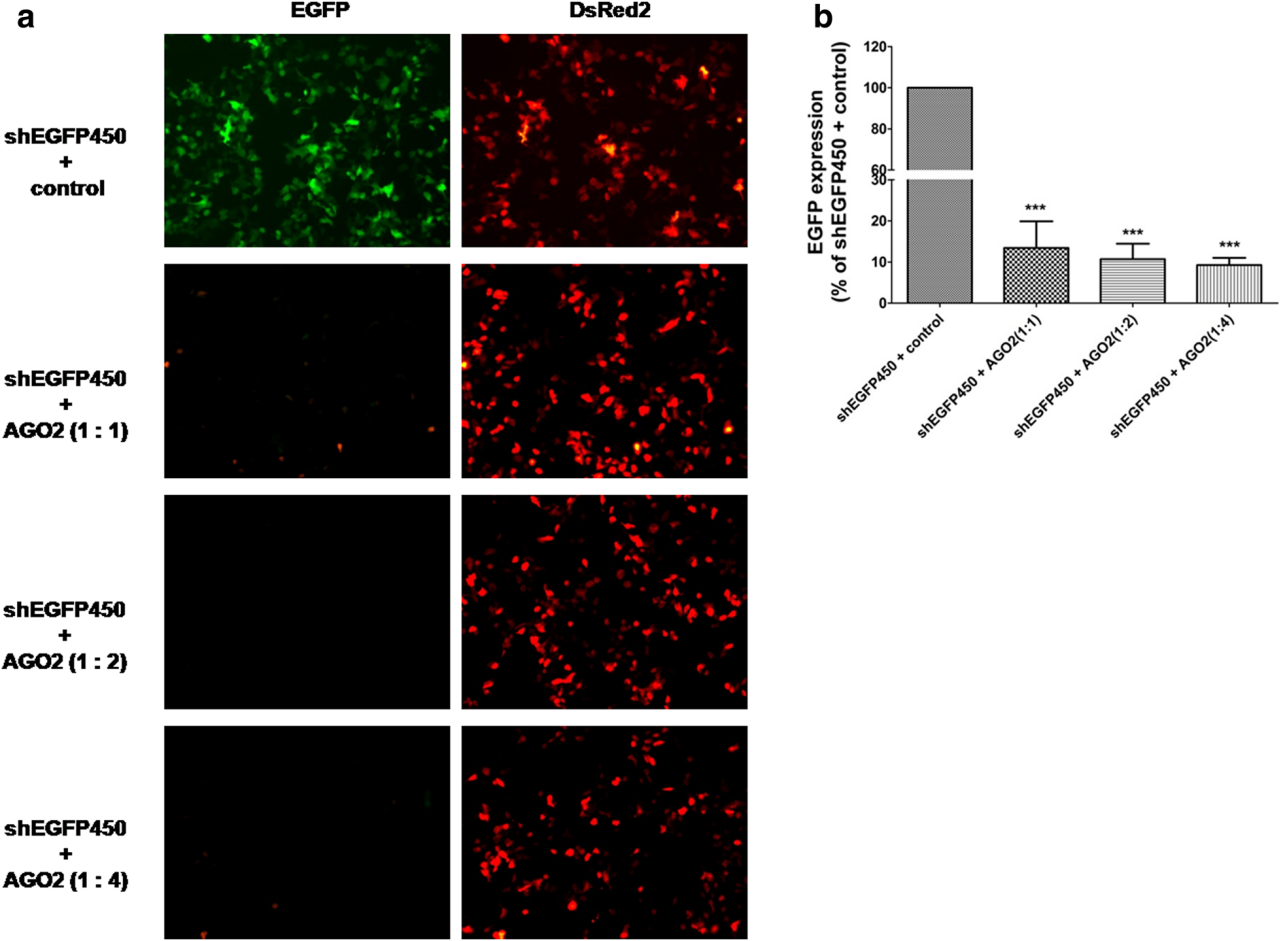
Dose-dependent analysis of AGO2 overexpression on RNAi efficiency. **a** Dose-dependent effects of AGO2 overexpression on EGFP silencing. HEK293 cells were co-transfected with pENTR/CMV-EGFP, pDsRed2-c1, shEGFP450 and different amount of AGO2 overexpression vector or the control vector without AGO2 insert. Representative *images* were shown from the cells 2 days after transfection; **b** the effect of AGO2 overexpression on EGFP silencing activities was quantified by analyzing G/R fluorescence intensity ratio. The results shown are mean ± SD from four independent experiments. ***P < 0.001 vs. control

Next, we evaluated the effects of the ratio between shRNA and AGO2 overexpression vectors on silencing efficiency. We co-transfected 100 ng of ineffective pENTR/U6-shEGFP450 vector and various amounts of pIRESneo-FLAG/HA-AGO2 (100, 200, 400 ng) together with 50 ng of EGFP-expressing and 50 ng of DsRed2-expressing vectors, obtaining a shEGFP450 to AGO2 mass ratio of 1:1, 1:2 and 1:4. Normalized to the negative control, which was made by removing AGO2 insert from the original pIRESneo-FLAG/HA-AGO2 vector, the silencing efficiencies of shEGFP450 with various amounts of AGO2 were all significantly increased due to the co-transfection of AGO2 vector. Although the silencing activities can be further improved with an increasing amount of AGO2 (1:2 and 1:4 of shEGFP450 to AGO2), the minimal amount of AGO2 plasmid at the mass ratio of 1:1 was sufficient to improve the silencing activity significantly (Fig. 2). It also implied that co-expression of AGO2 and shRNA in a single vector might achieve a prominent effect of silencing.

### Effects of stem length, loop size, and antisense strand location on the efficacy of AGO2-shRNA

**Fig. 3:**
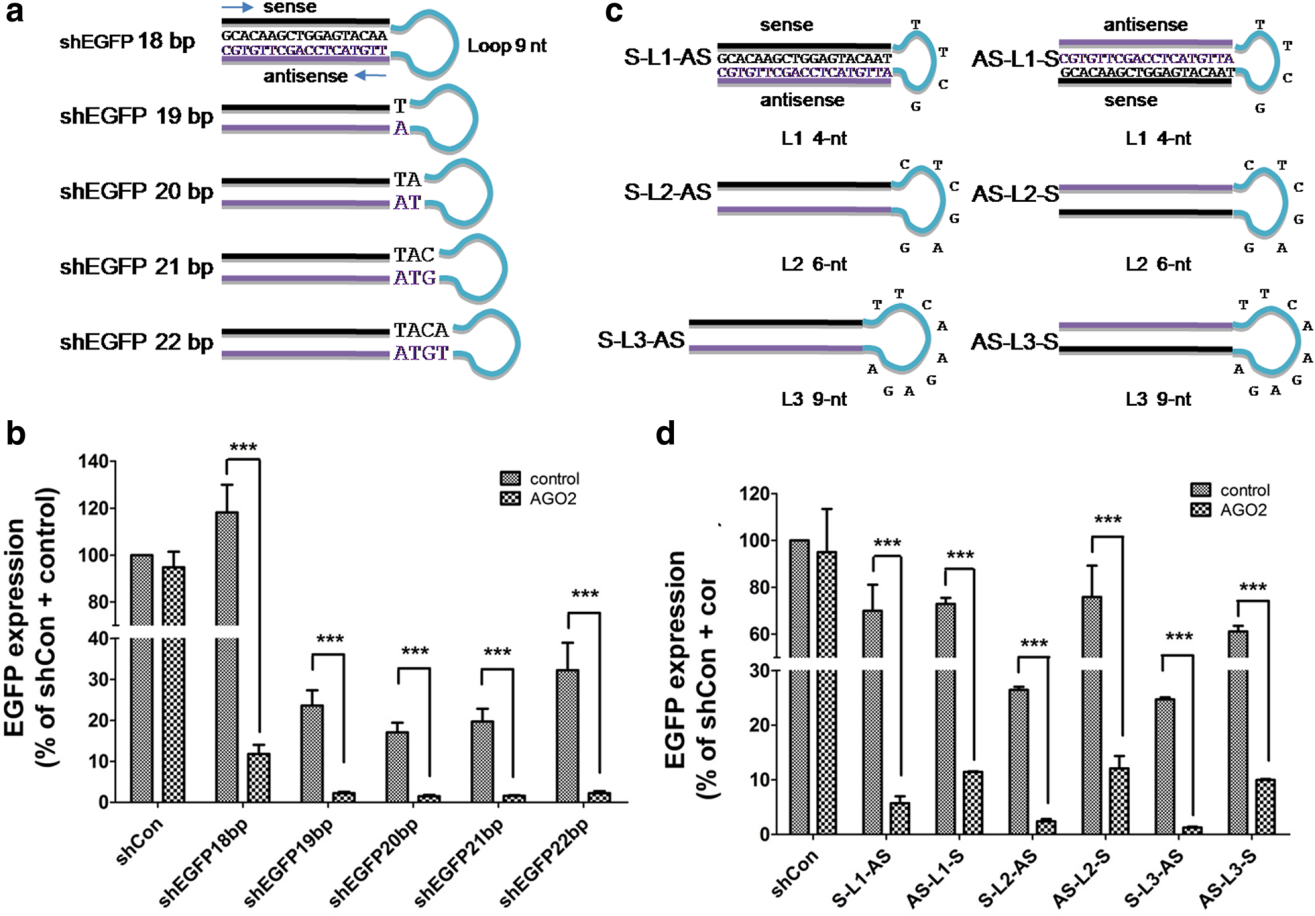
Effect of stem length, loop size, and antisense strand location of shRNA on RNAi efficiency. **a** Design of a set of shEGFP variants with different stem lengths. The stem lengths were indicated in the name of each shRNA. All the constructs were designed to target EGFP, starting at the position of 417 of EGPF with the stem sequence extending to the 3′ direction. The hairpin loop was the same (5′-TTCAAGAGA-3′) for all of the vectors; **b** comparison of the activities of shRNAs with different stem lengths in HEK293 cells. G/R fluorescence intensity ratio was measured from the cells co-transfected with pENTR/CMV-EGFP, pDsRed2-c1, shRNA vector with or without AGO2 overexpression vector. shCon/control group was used to normalize the silencing efficiency; **c** design of 19-bp hairpin stem of shEGFP417 at the orientation of Sense-Loop-Antisense (S-L-AS) or Antisense-Loop-Sense (AS-L-S) structure with three kinds of loop sequences, L1 (4-nt, TTCG), L2, (6-nt, CTCGAG) or L3 (9-nt, TTCAAGAGA); **d** comparison of the activities of S-L-AS and AS-L-S shRNA with different loop lengths in the presence or absence of AGO2 co-expression. ShCon/control group was used for the normalization of EGFP silencing activities. The results shown are mean ± SD from four independent experiments. ***P < 0.001

We evaluated the effects of AGO2 on the silencing efficiency of different shRNA structures. We designed a set of hairpins with different stem lengths, ranging from 18–22 bp (Fig. 3a). Compared to an unrelated control vector (shCon), the shRNAs alone with stem lengths between 19 and 22 bp showed 76, 83, 80 and 68 % inhibition (Fig. 3b). However, the shRNA with an 18 bp stem length showed no notable inhibitory activity. Overexpressing AGO2 significantly improved the silencing activities of all the shEGFP417 with different stem lengths (Fig. 3b). Consistent with the traditional shRNA design [24], the hairpins ranging from 19–21 bp in stem length produced better knockdown activities in our new design of AGO2/shRNA co-overexpression system.

To further improve the AGO2/shRNA system, we constructed several variants of shEGFP417 with different loop sizes (Fig. 3c). As shown in Fig. 3d, 19 bp stem length of shEGFP417 with 4-nt (TTCG), 6-nt (CTCGAG) or 9-nt (TTCAAGAGA) loop sequences exhibited different knockdown efficiency. We observed that the silencing efficiency of the shEGFP417 with 6-nt CTCGAG loop was comparable to that with the 9-nt TTCAAGAGA loop (>75 %). However, shEGFP417 with 4-nt loop sequence showed much less inhibition (~30 %). When AGO2 protein was co-expressed, the silencing efficiency of shEGFP417 with 4-, 6- or 9-nt loop were all improved dramatically.

According to the previous reports, silencing efficiency of shRNA was also influenced by the position of antisense sequences [25, 26]. We therefore constructed 19 bp stem length of shEGFP417 with three different sizes of loop at the orientation of antisense-loop-sense (AS-L-S) or sense-loop-antisense (S-L-AS) structures (Fig. 3c). When vectors expressing these shRNAs, EGFP/DsRed2 and AGO2 were co-transfected into HEK293 cells, we found that the silencing activities of shEGFP with 6- or 9-nt loop sequences in AS-L-S orientation were lower than the corresponding shRNA in S-L-AS orientation (Fig. 3d). Co-expression of AGO2 protein improved the silencing activities of AS-L-S shRNAs, but the efficiency was still lower than the respective AGO2-co-expressed traditional S-L-AS shRNA constructs. Overall, the results provide us a general guideline that 19–21-bp stem and 6- or 9-nt loop structure in the S-L-AS orientation should be used for the new AGO2-shRNA system.

### Effects of a single lentiviral vector co-expressing shRNA and AGO2 on silencing efficiency

**Fig. 4:**
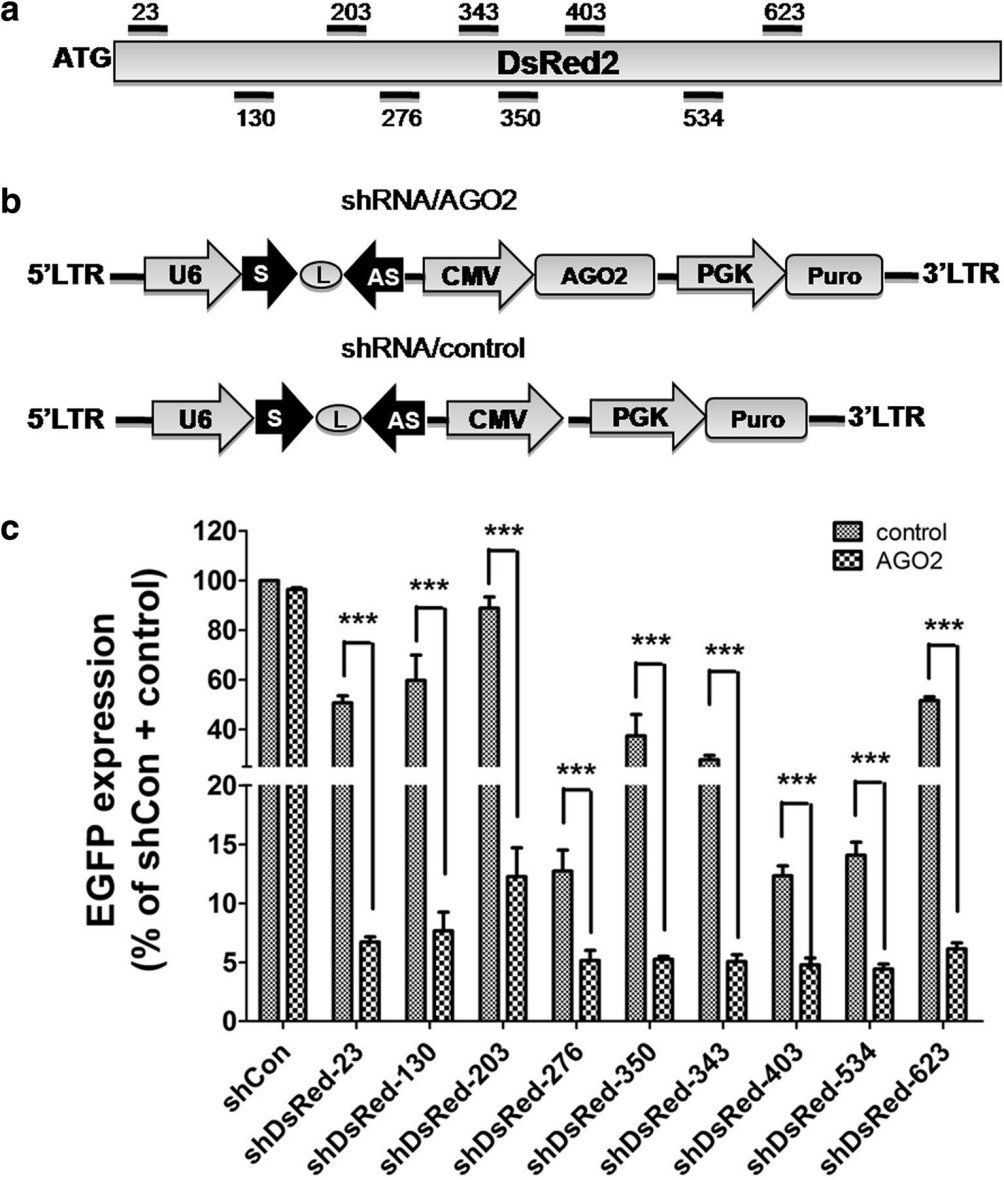
Co-expression of AGO2 enhances shRNA-mediated knockdown of DsRed2 florescence reporter. **a** Design of a set of shRNAs targeting DsRed2 coding region at different positions; **b** a schematic illustration of a shRNA lentiviral vector with CMV promoter-driven AGO2 (shRNA/AGO2). The corresponding control (shRNA/control) was generated by removing AGO2 fragment; **c** U6-driven shRNA with the co-expression of CMV-driven AGO2 or its control in the single plasmid were co-transfected with EGFP/DsReds plasmids into HEK293. Two days after transfection, R/G fluorescence intensity ratio was measured by flow cytometry analysis and the silencing efficiency was normalized to the shCon/control group. The results shown are mean ± SD from four independent experiments. ***P < 0.001

The aim of the studies in this section is to create a lentiviral vector that allows co-expression of shRNA and AGO2 using a single vector for a convenient shRNA cloning. To evaluate the silencing efficiency of a single vector co-expressing shRNA and AGO2, we applied the Dharmacon on-line siRNA design software to DsRed2 coding region and randomly chose nine siRNA sequences (Fig. 4a). For each shRNA, a single lentiviral vector harboring U6-shRNA and CMV-AGO2 expression cassettes was constructed as shown in Fig. 4b. For comparison, the respective U6-shRNA alone lentiviral vectors were generated by removing the AGO2 fragments. Nine shRNA/AGO2 or shRNA/control vectors were co-transfected with *EGFP/DsRed2* plasmids into HEK293 cells cultured in 24-well plates. Forty-eight hous after transfection, fluorescence intensity was visualized by fluorescence microscopy, followed by flow cytometry analysis. Without AGO2, shDsRed2-276, -403, -534 and -350 achieved over 70 % knockdown while shDsRed2-23, -130, -343 and -623 had moderate silencing activities (41–63 %); the shDsRed2-203 displayed the lowest silencing activity (11 %) (Fig. 4c). In contrast, with the shRNA/AGO2 vectors, all shDsRed2 exhibited significant knockdown of DsRed2 protein and the least effective shDsRed2-203 achieved an 88 % suppression of DsRed2 (Fig. 4c). These results indicate that RNAi activity is at least in part dependent on the co-expression of AGO2 protein in cells.

**Fig. 5:**
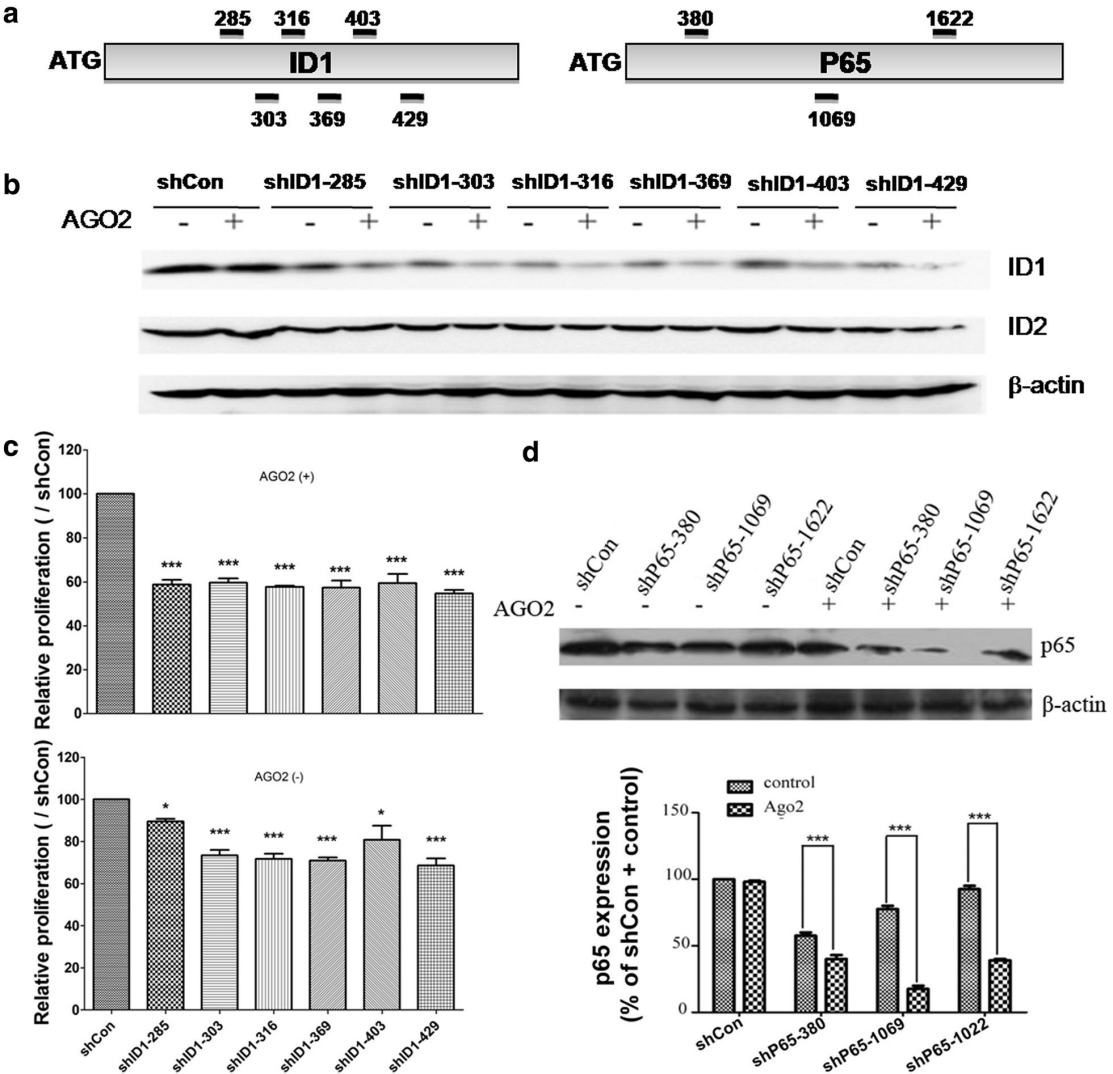
Co-expression of AGO2 enhances shRNA-mediated knockdown of endogenous genes. **a** Design of a set of shRNAs targeting human ID1 or P65 mRNA at different regions. The *number* indicates the initial position of siRNA in the coding region of genes; **b** a lentivirus harboring U6-driven shID1 and CMV-driven AGO2 or its control was used to infect HeLa cells twice and selected with puromycin for 1 week. Representatives of Western blots using anti-ID1 or anti-ID2 antibodies were shown. β-actin was used as a loading control; **c** HeLa cell proliferation after silencing of ID1. The same number of stably selected HeLa cells expressing shRNA with or without AGO2 protein were seeded on 96-well plates at a density of 1 × 10^4^ cells per well. After 24 h culture, cell proliferation was determined. The results shown are mean ± SD from four independent experiments. *P < 0.05 vs shCon, ***P < 0.001 vs shCon; **d** Western blot showing the knockdown of P65 in HeLa cells with a lentivirus-based shRNA with or without AGO2 co-expression. shCon was used as a negative control. β-actin was used as a loading control

Given the effectiveness of AGO2 in enhancing RNAi, we tested the shRNA/AGO2 vector for suppressing endogenous proteins. Inhibitor of Differentiation (ID1), an important helix-loop-helix (HLH) transcription factor involved in the proliferation and progression of many cancer types, was selected for this purpose. We selected six siRNA sequences targeting human ID1 coding region at different positions (Fig. 5a) and constructed lentiviral vectors expressing each shRNA with or without AGO2 co-expression. HeLa cells were infected with the lentivirus and the infected cells were selected using puromycin to assure complete infection. Without AGO2 co-expression, shID1-316, shID1-369 and shID1-429 displayed ~70 % knockdown of endogenous ID1 protein, shID1-303; shID1-403 exhibited ~60 % silencing while shID1-285 suppressed only ~30 % of ID1 compared to the shCon vector. (Fig. 5b). With AGO2 co-expression, all shID1 achieved over 70 % knockdown. However, all the shID1-AGO2 vectors had no effect on the expression of ID2, another member of ID family (Fig. 5b), indicating the specific silencing of shRNA-AGO2 system.

Functionally, we examined the effect of ID1 knockdown on cell proliferation and observed that all shID1-AGO2 infected HeLa cells had a lower cell proliferation rate compared to each corresponding shID1 without AGO2 co-expression (Fig. 5c).

P65 was selected as another target for the shRNA-AGO2 system and three shRNAs were chosen (Fig. 5a). As shown in Fig. 5d, without AGO2 co-expression, shP65-380 and shP65-1069 exhibited a moderate silencing efficiency while shP65-1622 was less effective. However, the co-expression of AGO2 significantly improved the knockdown efficiency, further supporting that AGO2 plays an essential role in shRNA-mediated gene silencing.

### A shRNA lentiviral vector with inducible AGO2 expression

**Fig. 6:**
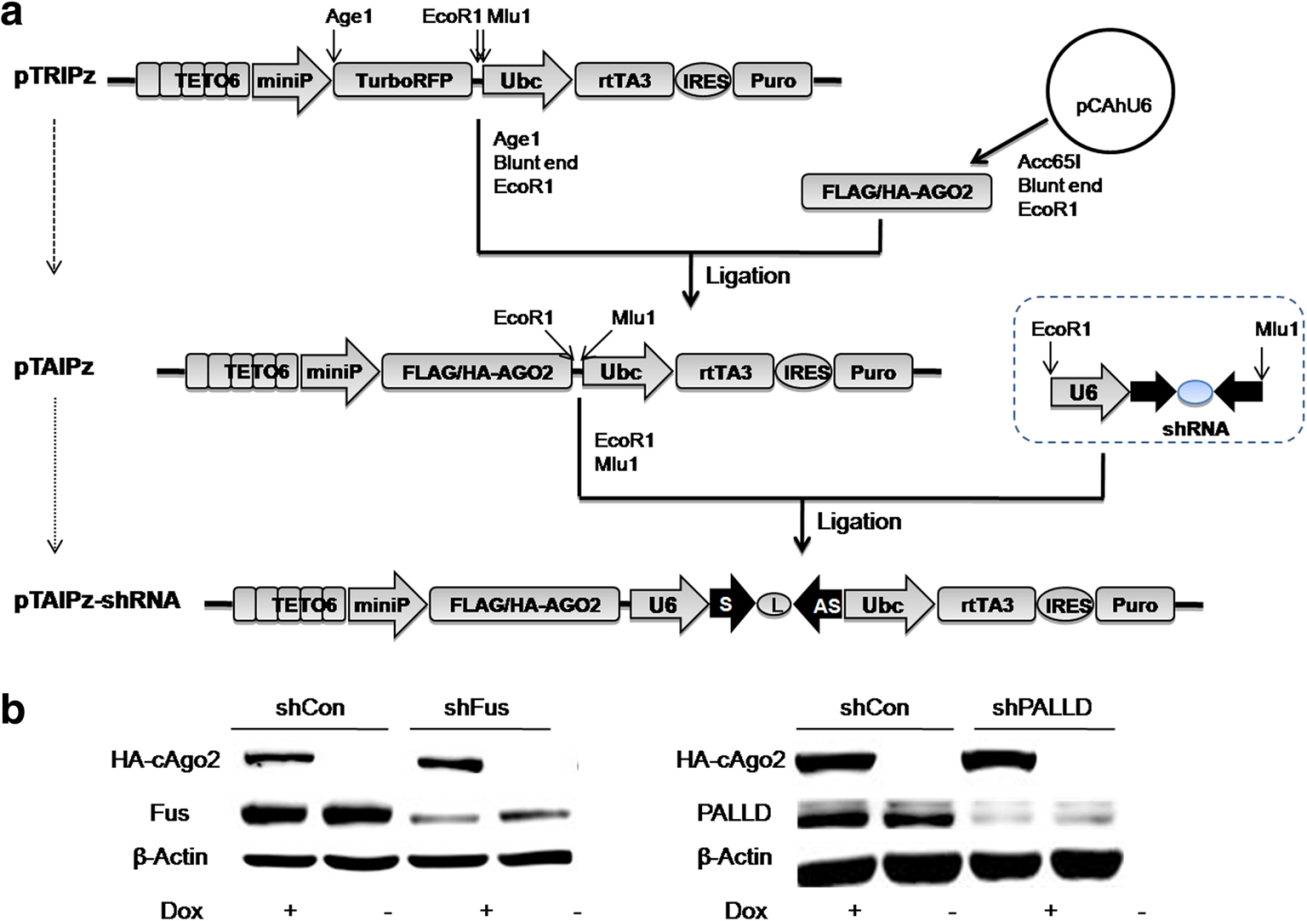
Enhancement of shRNA efficiency achieved by DOX inducible AGO2 overexpression lentiviral vector. **a** A schematic illustration of pTAIPz-shRNA lentiviral vector co-expressing U6-driven shRNA and inducible AGO2 under control of the tetracycline-inducible TETO6 promoter; **b** comparison of two target genes knockdown in A549 cells infected with lentivirus expressing shRNA with or without AGO2 induction. β-actin served as the loading control

The virus titers from the CMV promoter-driven AGO2 vector were extremely low (<10^3^ pfu/ml), which significantly limited its application. This is likely due to negatively impact of the package of lentivirus by the constitutive overexpression of FLAG/HA/AGO2. To overcome this difficulty, we generated a new shRNA lentiviral vector (TAIPz-shRNA) inductively expressing AGO2, which consists of U6-driven shRNA, TETO6 inducible promoter-drived FLAG/HA/AGO2 and Ubc promoter-drived rtTA3 transactivator (Fig. 6a). The virus titer packaged from the pTAIPz-shRNA was significantly increased over 1000 times (up to 10^6^pfu/ml) compared to the constitutive overexpression of AGO2. Next, we analyzed the shRNA knockdown efficiencies against Fus and PALLD targets in the infected cells with or without AGO2 induction. The expression of both targets were significantly reduced even without AGO2 induction. Similar to the results above, the silencing efficiencies of shFus and shPALLD were further enhanced by the induction of AGO2 expression in the presence of DOX (Fig. 6b). The successful induction of FLAG/HA/AGO2 was confirmed by Western blot using anti-HA antibody (Fig. 6b). The shRNA lentiviral vector with inducible AGO2 overexpression may serve as an additional tool for RNAi research in case of inhibitory shRNA sequences with low efficiency.

## Discussion

RNAi-based siRNA/shRNA has been developed as a robust tool for the inhibition of gene expression in vitro and in vivo. Nevertheless, the uncertainty of RNAi silencing efficiency is still a restriction of its application. As a key component of the RNA-induced silencing complex (RISC), AGO2 is responsible for the endonuclease cleavage of target mRNAs [20]. The process of RNAi cannot be accomplished without AGO2. Several studies have shown that the overexpression of AGO2 significantly improves shRNA-mediated gene silencing. Diederichs et al. co-transfected HEK293 cells with vectors expressing AGO2 and shEGFP and showed that AGO2 overexpression significantly enhanced the silencing efficiency of shEGFP [6]. Chen et al. found co-expression of AGO2 enhanced shRNA-induced RNAi in Xenopus CNS Neurons in vivo [19]. Similarly, Grimm et al. demonstrated that AGO2 obviously enhanced the inhibition efficiency and extended the inhibition time of shRNAs in mice. It also reduced the liver toxicity caused by shRNA [18]. Recently, the distinctive AGO2 property has been further exploited by constructing an Adeno-associated viral vector co-expressing AGO2 and shRNA [22]. They also showed that AGO2-overexpressing cell lines generated through lentiviral transduction yielded a better siRNA inhibition outcome and improved RNAi phenotypes. But they did not demonstrate the successful application of a single lentiviral vector co-expressing shRNA and AGO2.

In this study, we found that the overexpression of AGO2 strongly increased silencing efficiency of shRNAs. This effect was observed not only in U6-driven traditional shRNAs, but also in miR-30a-based shRNAmir30. In contrast, overexpression of AGO1, DICER or XPO5 did not exhibit significant improvement of RNAi activities even though they are essential in transporting or processing precursor dsRNAs.

As the knockdown efficiency of shRNAs is affected by the length of stem, the loop sequences and the location of antisense strand in shRNA structure [7, 27], we evaluated the effects of these factors in the new designed AGO2/shRNA co-expression system. Similar to traditional shRNAs, a hairpin ranging from 19–21 bp with 6–9 nt loop produces a better knockdown activity in the AGO2/shRNA system. In contrast to the previous report showing that the artificially synthesized AS-L-S shRNA outshined S-L-AS shRNA in gene silencing [28], our data indicate that S-L-AS shRNA was much more efficient than AS-L-S shRNA in gene silencing. This is also the case when the shRNA structures were co-expressed with AGO2, even though silencing efficiencies of both shRNA structures were dramatically increased by the overexpression of AGO2. The detailed mechanism of the different inhibition efficiencies driven by these two types of shRNA remains unclear. But, the discrepancy could be due to different genes that are targeted.

Based on the evaluation of shRNAs targeting either exogenous DsRed2 or endogenous ID1 and P65 genes, we demonstrate that the co-expression of shRNA and AGO2 in a single vector achieved a robust gene silencing outcome. Vector-based shRNA has its limitation due to the low transfection efficiency in many types of cells, poor integration ability, and insufficient expression level. Lentiviral vectors are a promising tool for both in vivo and ex vivo gene therapy as well as their extensively application in basic biomedical research because of their ability to incorporate into genomic DNA with high efficiency, especially in cells that are not actively dividing. The approach of lentivirus-based shRNA/AGO2 makes it possible in improving the shRNA-mediated silencing of the target mRNA, which provides a promising tool for RNAi application. Similar strategy should be also useful for miRNA overexpression in mammalian cells.

When we packaged lentivirus in 293T cells using a lentiviral vector with U6-driven shRNA and CMV-driven AGO2 expression cassettes, we observed that the lentivirus titer was extremely low. Initially, we thought that the low titer might be caused by the over-limited size of AGO2 insert in the lentiviral vector. However, when we removed the PGK-Puro expression cassette, we did not observe any improvements in virus titer. This suggests that the low virus titer is probably due to constitutive expression of AGO2. Indeed, the virus titer from inducible AGO2-shRNA vector was increased by over 1000 fold. This new inducible AGO2-shRNA lentiviral expression vector contains the TETO6-mini promoter-driven AGO2, Ubc promoter-driven rtTA3 and U6-shRNA expression cassette. When AGO2 was induced by the addition of Dox, the RNAi efficiency was significantly improved. The modified strategy of constitutive expression of shRNA and inducible expression of AGO2 in a single lentiviral-vector has advantages of a low toxicity and prolonged efficacy.

## Conclusions

We reported a novel lentiviral vector with an optimal design of shRNA and inducible AGO2 overexpression which has a robust silencing of exogenous and endogenous targets in mammalian cells and provides a powerful tool for RNAi application.

## Methods

### Plasmids and vector construction

pIRESneo-FLAG/HA-AGO1, pIRESneo-FLAG/HA-AGO2, pDEST-myc-Dicer, and pkmyc-XPO5, overexpressing human AGO1, AGO2, DICER or EXPORTIN-5 (XPO5) were purchased from Addgene. pIRESneo-FLAG/HA-Control generated from pIRESneo-FLAG/HA-AGO2 by removing the AGO2 sequence was used as a negative control for AGO2 experiment. The codon-optimized human AGO2 with FLAG/HA tags in pCAhU6 vector was kindly provided by Dr. Dirk Grimm (University of Heidelberg). pDsRed2-c1 vector expressing red florescence protein was purchased from Clontech. pENTR/CMV-EGFP vector expressing enhanced green florescence protein (EGFP) was generated as previously described [10].

Human U6 promoter-driven shRNAs were constructed using linearized BLOCK-iT™ U6 RNAi Entry Vector (Invitrogen). Two siRNA sequences with different silencing activities against EGFP coding region at position of 417-437 (5′-GCA CAA GCT GGA GTA CAA CTA-3′) and 450-470 (5′-CGT CTA TAT CAT GGC CGA CAA-3′) were selected for constructing EGFP silencing vectors, named pENTR/U6-shEGFP417 and pENTR/U6-shEGFP450. pSM2c-shEGFP417miR30 (Openbiosystem) was a miR-30-based shEGFP417 vector targeting EGFP at the same position of 417-437.

To construct lentiviral vectors co-expressing shRNAs and AGO2, the pLVX-puro vector (Clontech) was selected as a backbone plasmid. Since there are two *BsmB*I sites located in PGK promoter and puromycin resistant selection gene in the pLVX-puro vector, we removed both *BsmB*I sites via mutagenesis using overlap PCR strategy. The *BsmB*I site in the PGK promoter was changed to CGCCTC, which did not affect the promoter activity (data not shown). The *BsmB*I site in the puromycin resistant selection gene was changed to CGTGTC without affecting the encoded amino acid. The modified pLVX-puro was then digested with *Cla*I and *Xba*I and used for cloning U6 and CMV-AGO2 expression cassettes with Gibson Assembly kit (New England Biolab). Briefly, U6 promoter was PCR-amplified with the forward primer (5′-GCA GAG ATC CAG TTT ATC GAA CAA TCG ATA AGG TCG GGC AGG A-3′) and reverse primer (5′-CAA TGT CAA CGC GTA AAA AAG AGA CGC GTC TCA CGG TGT TTC GTC CTT TC-3′). Two *BsmB*I sites (underlined) in the reverse primer were included for cloning shRNA inserts. CMV-FLAG/HA/AGO2 fragment was amplified with the forward primer (5′-TTT TTT ACG CGT TGA CAT TGA TTA TTG ACT AGT TAT TAA TAG-3′) and reverse primer (5′-CTA CCC GGT AGA ATT ATA TAG CGA TCC ACT GAA TTC-3′) using a codon-optimized AGO2 vector of pCAnU6 as a template. We inserted a ccdB fragment with corresponding *BsmB*I site into the assembled vector, named pLVX/U6-ccdB-CMV-AGO2, which can be further digested by *BsmB*I, leaving five overhangs of 5′-CGGT and 5′-TTTT for the ligation of an annealed shRNA insert with 5′ overhangs of 5′-ACCG and 5′-AAAA.

To construct an inducible AGO2 lentiviral vector, the TurboRFP fragment in pTRIPz control vector (Openbiosystems) was first digested with *Age*I, blunt-end with Klenows in the presence of dNTP, and then digested with *EcoR*I. The insert of AGO2 with FLAG/HA tag at the N-terminus was released from the pCAhU6 vector by *Acc65*I digestion, blunt-end with Klenows, and then digested with *EcoR*1. The new construct of pTAIPz consists of 6 repeats of a 19 bp tet operator sequence, followed by a minimal CMV promoter (miniP) and FLAG/HA/AGO2 insert. The co-expression rtTA3 transactivator and IRES-based puromycin is controlled by the human ubiquitin C (Ubc) promoter.

To generate a single lentiviral vector co-expressing shRNA and inducible AGO2, the U6-shRNA expression cassette released from pLVX-U6-shRNA-CMV-AGO2 was subcloned into the pTAIPz vector through *Eco*RI-*Mlu*I sites, resulting in a pTAIPz-shRNA construct.

### Mammalian cell culture and fluorescence reporter assays

HEK293, HeLa, and A549 cells were obtained from American Type Culture Collection (ATCC) and were grown in DMEM medium with 10 % fetal bovine serum (FBS). The fluorescence reporter assays were performed in 24-well plates. In brief, cells were seeded at a density of 6 × 10^4^ cells per well. After overnight incubation, the cells were co-transfected with different combinations of plasmids using linear polyethylenimine (PEI) transfection reagent. Six h after transfection, the cells were changed to fresh DMEM medium containing 10 % FBS and incubated for additional 48 h. The fluorescence of cells were first observed and photographed under an inverted fluorescence microscope, then trypsinized, and collected for flow cytometry analysis. The averaged green to red (G/R) or red to green (R/G) fluorescence ratio was used as an index for RNAi efficiency of EGFP or DsRed2 knockdown.

### Lentivirus package and transduction

Lentiviral particles were packaged in 293T cells by transfection of three individual plasmids at a ratio of 3:1:3: (1) psPAX2 encoding HIV Gag-Pol (Addgene, 12260), (2) pVSVg (Addgene, 8454) encoding VSV-G glycoprotein, and (3) a lentiviral vector. Briefly, 293T cells were seeded on 10 cm culture dishes at a density of 4 × 10^6^ cells per dish. After 24 h incubation, the cells were transfected with lentiviral vector (6.9 µg) and packing plasmids (17.1 µg) using PEI reagents. Cell-free supernatants were harvested 48 and 72 h after transfection and used for subsequent cell infection in the presence of 8 ng/ml polybrene. The infected cells were selected by supplementing culture medium with 1–2 µg/ml puromycin after 48 h infection.

### Cell proliferation assay

A lentivirus expressing shRNA against ID1 or an un-related control shRNA with or without AGO2 co-expression were used to infect HeLa cells in the presence of polybrene. After 48 h infection, the infected cells were selected by supplementing culture medium with 1.5 µg/ml puromycin. Cell proliferation was then determined using the CellTiter 96 Aqueous One Solution proliferation assay (Promega).

### Western blot analysis

Cells were lysed with ice-cold RIPA buffer (50 mM Tris–HCl, pH 7.5; 150 mM NaCl; 1 % NP-40; 0.25 % sodium deoxycholate, 1 mM EDTA), supplemented with a protease inhibitor cocktail (Roche). Protein concentration was determined using Bradford protein assay kit (Bio-Rad). Equal amounts of extracts were then electrophoresed on SDS–polyacrylamide gel and electro-blotted to nitrocellulose filter membranes (Millipore). Membranes were immersed in blocking buffer (5 % degreased milk powder) for 1 h and incubated with primary antibodies. The primary antibodies used are as follows: rabbit monoclonal anti-ID1, -ID2 (Cat #MA201, #MA229, CalBioreagents, San Mateo, CA, USA; 1:1000 dilutions); rabbit polyclonal anti-P65 (Cat #SC-372, Santa Cruz, Santa Cruz, CA, USA, 1:800 dilutions), rabbit polyclonal anti-Fus, anti-PALLD, anti-beta-actin and anti-HA (Proteintech Group, 1:2000 dilutions). After rinsing, membranes were incubated with horseradish peroxidase-conjugated secondary antibody at dilutions of 1:10,000 for 1 h at room temperature. Immunoreactive bands were detected by exposing to X-ray film.

### Statistical analysis

All data shown are mean values of at least four experiments, each performed in triplicate, with standard deviation (SD). Statistically significant differences between groups were evaluated using the SPSS software. One asterisk, double asterisk, and three asterisks represent a significant difference between experimental pairs of p < 0.05, 0.01, and 0.001, respectively.

## References

[1] Fire A, Xu S, Montgomery MK, Kostas SA, Driver SE, Mello CC (1998). Potent and specific genetic interference by double-stranded RNA in Caenorhabditis elegans. Nature.

[2] Perkel JM (2012). RNAi therapeutics: the teenage years. Biotechniques.

[3] Maczuga P, Lubelski J, van Logtenstein R, Borel F, Blits B, Fakkert E, Costessi A, Butler D, van Deventer S, Petry H (2013). Embedding siRNA sequences targeting apolipoprotein B100 in shRNA and miRNA scaffolds results in differential processing and in vivo efficacy. Mol Ther J Am Soc Gene Ther.

[4] Berns K, Hijmans EM, Mullenders J, Brummelkamp TR, Velds A, Heimerikx M, Kerkhoven RM, Madiredjo M, Nijkamp W, Weigelt B (2004). A large-scale RNAi screen in human cells identifies new components of the p53 pathway. Nature.

[5] Dietzl G, Chen D, Schnorrer F, Su KC, Barinova Y, Fellner M, Gasser B, Kinsey K, Oppel S, Scheiblauer S (2007). A genome-wide transgenic RNAi library for conditional gene inactivation in Drosophila. Nature.

[6] Diederichs S, Jung S, Rothenberg SM, Smolen GA, Mlody BG, Haber DA (2008). Coexpression of Argonaute-2 enhances RNA interference toward perfect match binding sites. Proc Natl Acad Sci USA.

[7] Paddison PJ, Cleary M, Silva JM, Chang K, Sheth N, Sachidanandam R, Hannon GJ (2004). Cloning of short hairpin RNAs for gene knockdown in mammalian cells. Nat Methods.

[8] Elmen J, Thonberg H, Ljungberg K, Frieden M, Westergaard M, Xu Y, Wahren B, Liang Z, Orum H, Koch T (2005). Locked nucleic acid (LNA) mediated improvements in siRNA stability and functionality. Nucleic Acids Res.

[9] Li L, Lin X, Khvorova A, Fesik SW, Shen Y (2007). Defining the optimal parameters for hairpin-based knockdown constructs. RNA.

[10] Gou D, Weng T, Wang Y, Wang Z, Zhang H, Gao L, Chen Z, Wang P, Liu L (2007). A novel approach for the construction of multiple shRNA expression vectors. J Gene Med.

[11] Silva JM, Li MZ, Chang K, Ge W, Golding MC, Rickles RJ, Siolas D, Hu G, Paddison PJ, Schlabach MR (2005). Second-generation shRNA libraries covering the mouse and human genomes. Nat Genet.

[12] Hwang SK, Chang SH, Minai-Tehrani A, Kim YS, Cho MH (2013). Lentivirus-AIMP2-DX2 shRNA suppresses cell proliferation by regulating Akt1 signaling pathway in the lungs of AIMP2(+)/(−) mice. J Aerosol Med Pulm Drug Deliv.

[13] Gou D, Narasaraju T, Chintagari NR, Jin N, Wang P, Liu L (2004). Gene silencing in alveolar type II cells using cell-specific promoter in vitro and in vivo. Nucleic Acids Res.

[14] Shan G, Li Y, Zhang J, Li W, Szulwach KE, Duan R, Faghihi MA, Khalil AM, Lu L, Paroo Z (2008). A small molecule enhances RNA interference and promotes microRNA processing. Nat Biotechnol.

[15] Liu J, Carmell MA, Rivas FV, Marsden CG, Thomson JM, Song JJ, Hammond SM, Joshua-Tor L, Hannon GJ (2004). Argonaute2 is the catalytic engine of mammalian RNAi. Science.

[16] Parker GS, Maity TS, Bass BL (2008). dsRNA binding properties of RDE-4 and TRBP reflect their distinct roles in RNAi. J Mol Biol.

[17] Yi R, Qin Y, Macara IG, Cullen BR (2003). Exportin-5 mediates the nuclear export of pre-microRNAs and short hairpin RNAs. Genes Dev.

[18] Grimm D, Wang L, Lee JS, Schurmann N, Gu S, Borner K, Storm TA, Kay MA (2010). Argonaute proteins are key determinants of RNAi efficacy, toxicity, and persistence in the adult mouse liver. J Clin Investig.

[19] Chen CM, Chiu SL, Shen W, Cline HT (2009). Co-expression of Argonaute2 enhances short hairpin RNA-induced RNA interference in xenopus CNS neurons in vivo. Front Neurosci.

[20] Hutvagner G, Simard MJ (2008). Argonaute proteins: key players in RNA silencing. Nat Rev Mol Cell Biol.

[21] Lund E, Sheets MD, Imboden SB, Dahlberg JE (2011). Limiting Ago protein restricts RNAi and microRNA biogenesis during early development in Xenopus laevis. Genes Dev.

[22] Borner K, Niopek D, Cotugno G, Kaldenbach M, Pankert T, Willemsen J, Zhang X, Schurmann N, Mockenhaupt S, Serva A (2013). Robust RNAi enhancement via human Argonaute-2 overexpression from plasmids, viral vectors and cell lines. Nucleic Acids Res.

[23] Xia XG, Zhou H, Samper E, Melov S, Xu Z (2006). Pol II-expressed shRNA knocks down Sod2 gene expression and causes phenotypes of the gene knockout in mice. PLoS Genet.

[24] Sui G, Soohoo C, el Affar B, Gay F, Shi Y, Forrester WC, Shi Y (2002). A DNA vector-based RNAi technology to suppress gene expression in mammalian cells. Proc Natl Acad Sci USA.

[25] Dallas A, Johnston BH (2013). Design and chemical modification of synthetic short shRNAs as potent RNAi triggers. Methods Mol Biol.

[26] Ge Q, Ilves H, Dallas A, Kumar P, Shorenstein J, Kazakov SA, Johnston BH (2010). Minimal-length short hairpin RNAs: the relationship of structure and RNAi activity. RNA.

[27] Brummelkamp TR, Bernards R, Agami R (2002). A system for stable expression of short interfering RNAs in mammalian cells. Science.

[28] Dallas A, Ilves H, Ge Q, Kumar P, Shorenstein J, Kazakov SA, Cuellar TL, McManus MT, Behlke MA, Johnston BH (2012). Right- and left-loop short shRNAs have distinct and unusual mechanisms of gene silencing. Nucleic Acids Res.

